# FSIP1 Is Associated with Poor Prognosis and Can Be Used to Construct a Prognostic Model in Gastric Cancer

**DOI:** 10.1155/2022/2478551

**Published:** 2022-06-03

**Authors:** Xiuchun Yan, Junzhu Dai, Ying Han, Qi You, Yao Liu

**Affiliations:** ^1^Department of Gastroenterological Surgery, Harbin Medical University Cancer Hospital, Harbin Medical University, Harbin 150081, China; ^2^Department of Pain Medicine, Harbin Medical University Cancer Hospital, Harbin 150081, China; ^3^Department of Cancer Prevention and Physical Examination Center, Harbin Medical University Cancer Hospital, Harbin 150081, China

## Abstract

Gastric cancer (GC) is one of the most common upper gastrointestinal malignant tumors, and the incidence of the GC shows an increasing trend in the past years. Finding more sensitive markers will help to reveal the mechanism of GC progression and clinic diagnoses. This study first analyzed the mRNA expression level of FSIP1 in TCGA GC samples and the significance in predicting the prognosis. KEGG and GO analyses were used to explore the molecular mechanism of FSIP1 in GC progression. This study further retrospectively analyzed 166 clinical samples of GC from Harbin Medical University Cancer Hospital and evaluated the expression level of FSIP1 by immunohistochemistry. Kaplan-Meier and Cox multivariate analysis was used to investigate the prognostic value of FSIP1 expression in GC patients. We also identified correlations between FSIP1 and clinicopathological characteristics. This study found that the mRNA level of FSIP1 was significantly upregulated in GC compared with nontumor specimens and correlated with poor prognosis. Immunohistochemistry confirmed the results of bioinformatics analysis of the TCGA GC database. FSIP1 was associated with pTNM pathological stage, tumor location, and neural invasion. In addition, multivariate Cox regression analysis showed that FSIP1, T classification, and N classification were independent posterior factors of patients and could be combined with pathological features to construct a nomogram prognostic model. Overall, our results suggest that FSIP1 is expected to be an independent prognostic indicator of GC.

## 1. Introduction

Gastric cancer (GC) is a common malignant tumor with high morbidity and mortality globally. According to the 2022 World Cancer Statistics, there are more than 26,000 new cases and 11,000 deaths in America [1]. The morbidity rate of male is two times higher than that of female [2]. Although the morbidity rate of GC (combined cardia and noncardia cancers) has declined globally, it is worth noting that it increases in young patients (age < 50 years old) in low-risk and high-risk countries, including China, India, USA, Japan, and Russia [3, 4]. This indicates that the morbidity of GC has increased among the younger generation and the number of new cases is expected to continue to rise, highlighting that GC remains a major worldwide public health challenge.

Fibrous sheath interacting protein 1 (FSIP1) is a testicular antigen associated with spermatogenesis and it has multiple biological functions. Studies found that FSIP1 was necessary for normal spermatogenesis and played an important role in acrosome biogenesis and enflagellation by attenuating the function of intraflagellar transporters [5]. FSIP1 can significantly affect autophagy and inhibit mitochondrial function by upregulating AMP-activated protein kinase activity [6]. Furthermore, FSIP1 inhibits cell proliferation and induces apoptosis by inhibiting the PI3K/AKT pathway in bladder urothelial carcinoma [7]. Several studies also showed that FSIP1 was overexpressed in various tumor cells, including breast cancer, bladder cancer, non-small-cell lung cancer, and colon cancer [8–11]. Numerous studies indicated that FSIP1 was a key molecular node in the progression of these diseases and could be served as a novel potential prognostic marker and promising therapeutic target. Such as breast cancer, studies found that the expression of FSIP1 was positively correlated with HER2, recurrence, and metastasis but negatively correlated with survival probability [10]. Liu et al. [12] found that FSIP1 directly binded to the intracellular domain of HER2 and inhibited the expression of FSIP1 in HER2-positive breast cancer cells, resulting in decreased cell proliferation, increased apoptosis, and decreased cell migration and invasion capability through co-immunoprecipitation and microthermal atrophy. This suggests that FSIP1 may not only be a prognostic marker of breast cancer but also a potential drug therapy target.

However, the clinical significance of FSIP1 and its molecular biological function in GC is still unclear and deserves further study. This study first analyzed the relationship between the mRNA expression level of FSIP1 in TCGA-GC and prognosis. We also analyzed its possible molecular mechanism in the occurrence and development of GC. We further retrospectively analyzed the relationship among the immunohistochemical expression of FSIP1, clinicopathological characteristics, and prognosis in patients with GC admitted to Harbin Medical University Cancer Hospital and then constructed a prognostic model. The main purpose of this study is to explore the clinical significance of FSIP1 in GC and to provide a new possible biomarker for the clinical diagnosis, treatment, and prognosis evaluation.

## 2. Materials and Methods

### 2.1. Patient Characteristics

166 GC patients who underwent radical gastrectomy surgery in Harbin Medical University Cancer Hospital between May and July 2015 were included. Exclusion criteria are as follows: (1) Patients received preoperative neoadjuvant therapy; (2) died not due to GC; (3) with incomplete clinical data or missing important pathological data; and (4) combined with severe cardiovascular disease. All pathological tissues were diagnosed as gastric adenocarcinoma by several authoritative pathologists. TNM staging, histological type, and degree were according to AJCC 8^th^ edition GC staging system [13]. This research was performed in accordance with the Helsinki Declaration. All patients signed informed consent and they were informed about study. All 166 patients were reviewed every 3-6 months for serum tumor markers or imaging examination [computed tomography (CT), ultrasound, and gastroscopy].

### 2.2. Bioinformatics Analysis

The mRNA expression profile dataset was from the TCGA-GC dataset, (https://tcgadata.nci.nih.gov/tcga/). KEGG (Kyoto Encyclopedia of Genes and Genomes) and GO (Gene Ontology) pathway enrichment analyses were used for genome functional annotation. KEGG and GO analyses were performed using the Genomics Analysis and Visualization Platform tool (http://r2.amc.nl).

### 2.3. Immunohistochemistry

Paraffin sections of GC patients were sequentially deparaffinized with xylene and then dehydrated with ethanol. Then, we washed the sections with distilled water and pretreated with EDTA extract at pH 8.0 in a pressure cooker at 120°C for 2 min. Endogenous peroxidase was inhibited with 3.0% H_2_O_2_ for 45 min. Sections were incubated with goat serum (Boster, AR0009) for 1 hour at room temperature to block the no-specificity. The slices were then incubated with primary antibody at 4°C overnight and with secondary antibody at 37°C for 30 min on the second day. Rabbit Anti-FSIP1 antibody (Bioss, bs-8575R) was used as the primary antibody, and 2-step plus Poly-HRP Anti Mouse/Rabbit IgG Detection System (Elabscience, E-IR-R217) was used as the secondary antibody. After incubation with secondary antibodies, sections were washed in PBS for 15 min. The chromogenic reaction was carried out by diaminobenzidine (DAB) staining. Nuclei were stained with hematoxylin, dehydrated with alcohol, eluted with xylene, and mounted with neutral resin finally. The slices stained with immunohistochemistry were analyzed and processed by Image-Pro Plus software version 6.0.2 (Media Cybernetics, USA) [14].

### 2.4. Statistical Analysis

Overall survival (OS) was defined as the time from surgery to death. Disease-specific survival (DSS) was defined as the proportion of patients with GC who did not die of GC after a certain period of time. Progress-free survival (PFS) was defined as the time between the treatment and the observation of disease progression or death from any cause in patients with tumor disease. Data were presented as median and mean. Survival analysis was performed by the Kaplan-Meier method and tested by the Log-rank. The analysis of count data was performed by the chi-square test. Univariate and multivariate analyses based on Cox hazard regression models were used to evaluate the clinical factors and FSIP1 expression. Hazard ratio (HR) and 95% confidence interval (CI) were used as the effect factor. Prognostic model was constructed by using R studio 4.0.2. All statistical analyses were performed using SPSS 22.0 (SPSS, USA). Two-tailed *P* < 0.05 was considered statistically significant.

## 3. Results

### 3.1. Bioinformatics Analysis of FSIP1-Related Genes

**Figure 1:**
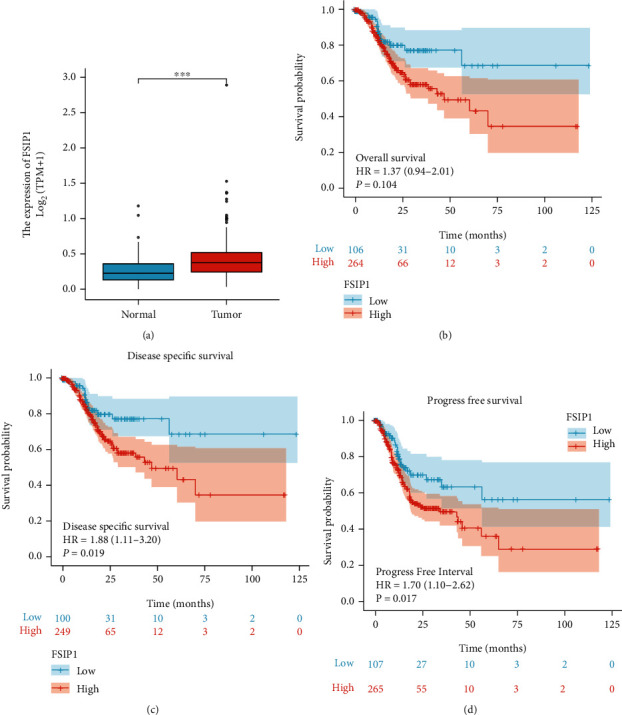
The mRNA expression level of FSIP1 in the TCGA GC dataset. (a) FSIP1 expression in tumors is higher than that in normal tissues (*P* < 0.001), (b–d) Overall Survival (*P* = 0.104), Disease Specific Survival (*P* = 0.019), and Progress Free Survival (*P* = 0.017) analysis according to the expression of FSIP1.

**Figure 2:**
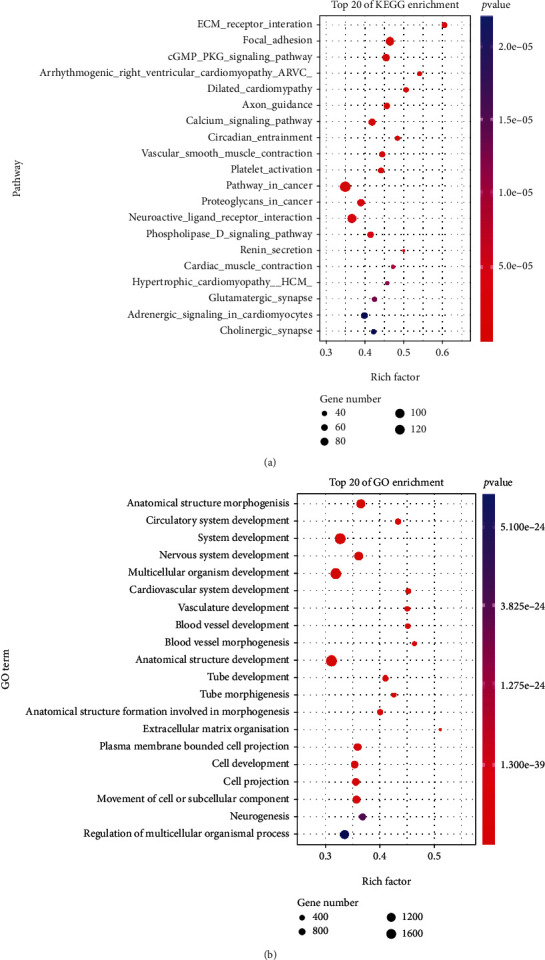
(a) KEGG enrichment analysis of genes associated with FSIP1 in the TCGA gastric cancer dataset. (b) GO enrichment analysis of genes associated with FSIP1 in the TCGA gastric cancer dataset.

The analysis in the TCGA-GC dataset showed that the mRNA expression level of FSIP1 was significantly higher in tumors than in normal control tissues (Figure 1(a)). Survival analysis found that although there was no statistically significant difference between FSIP1 and OS, patients with high expression of FSIP1 tended to have shorter OS (Figure 1(b)). In addition, high expression of FSIP1 was negatively correlated with DSS (Figure 1(c), *P* = 0.019) and PFS (Figure 1(d), *P* = 0.017). KEGG analysis showed that genes highly related to FSIP1 were mainly involved in biological processes such as ECM receptor interaction, cell signal transduction, vascular smooth muscle contraction, and platelet activation (Figure 2(a)). GO analysis showed that the biological functions of FSIP1 and its related genes mainly focused on biological processes such as anatomical morphogenesis, circulation, nerve, and vasculature development (Figure 2(b)). These biological behaviors may be involved in the progression of GC.

### 3.2. Immunohistochemical Expression of FSIP1 and Survival Probability of Patients

**Figure 3:**
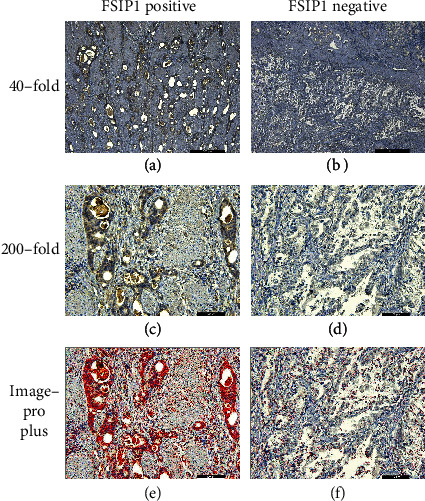
FSIP1 negative and positive immunohistochemical expression at 40-fold and 200-fold.

**Figure 4:**
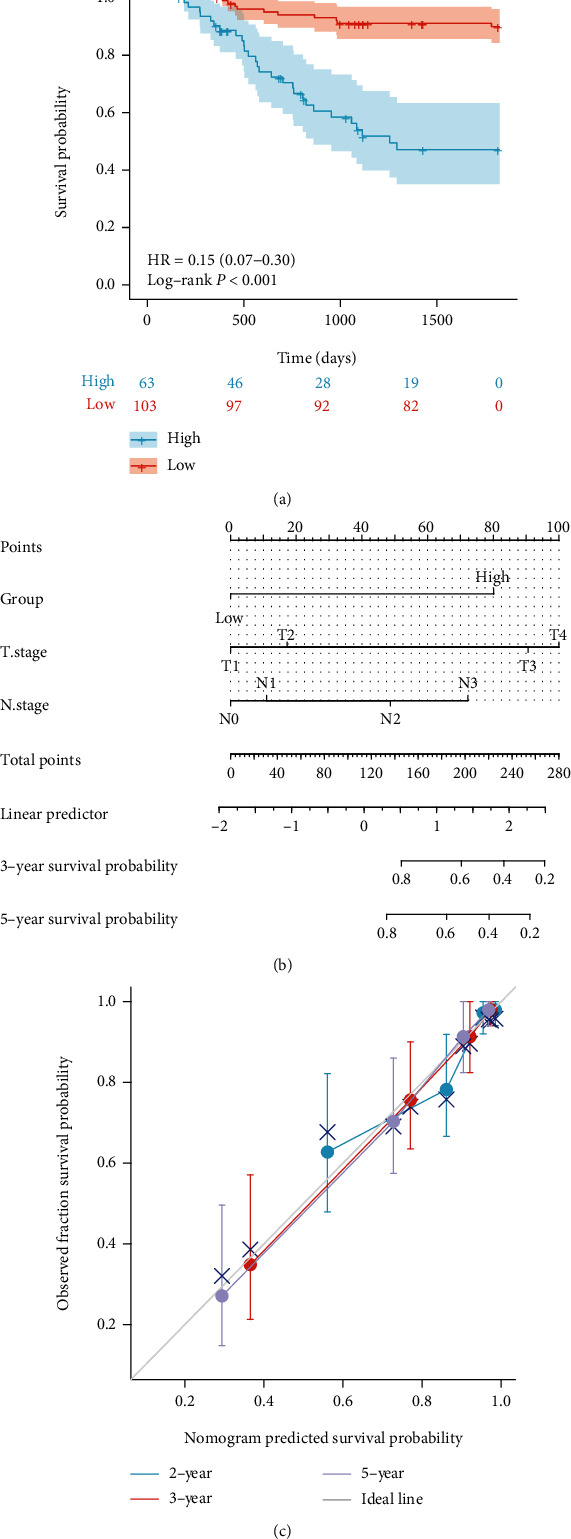
(a) FSIP1 immunohistochemical expression is associated with better prognosis (*P* < 0.001); (b) Nomogram prediction model of FSIP1 immunohistochemical expression combined with T classification and N classification; (c) Calibration analysis shows the C-index is 0.822(0.793-0.851).

FSIP1 is mainly expressed on the cell membrane of GC cells.  Figure 3 shows the representative FSIP1 immunohistochemical staining images (Figures 3(a) and 3(c), high expression of FSIP1 in GC; Figures 3(b) and 3(d) low expression of FSIP1 in GC). Figures 3(e) and 3(f) show the analysis of FSIP1 expression area by Image-Pro Plus software. The expression level of FSIP1 was quantified as positive area/total area, with an area ratio of 10.0% as the cut-off value. According to the results of statistical analysis, a total of 103 GC patients with an area percentage of ≤10.0% were defined as low-expression group; a total of 63 GC patients with an area percentage of >10.0% were defined as high-expression group. The mortality probability of patients with low expression was 21.36%, and that of patients with high expression was 69.84%. There was statistically significant difference in survival probability between the groups with different FSIP1 expression levels (*P* < 0.001 in Figure 4(a)).

### 3.3. The Relationship between the Expression of FSIP1 and Clinicopathological Characteristics

The average follow-up time was 46 months (5 to 60 months). There were thirty eight (22.9%) patients died in this research. Chi-square test was used to analyze the relationship between the expression of FSIP1 and clinicopathological characteristics. Patients with high expression of FSIP1 were correlated with tumor pathological stage (*P* < 0.001), tumor location (*P* = 0.003), and neural invasion (*P* = 0.012, Table 1). Cox regression was used to analyze the relationship between OS and clinicopathological characteristics including gender, age, T classification, N classification, body mass index (BMI), neutrophil-lymphocyte ratio (NLR), platelet-lymphocyte ratio (PLR), serum CD4^+^T lymphocytes, serum CD8^+^T lymphocytes, carcinoembryonic antigens CA19-9, CA72-4, CA125, tumor location, histological type, neural invasion, and vascular invasion (Table 2). Among these factors, the expressions of FSIP1 (HR 0.143, 95% CI 0.069-0.295, *P* < 0.001), T classification (HR 0.109, 95% CI 0.033-0.357, *P* < 0.001), N classification (HR 4.041, 95% CI 1.504-10.854, *P* = 0.006), CA19-9 (HR 1.002, 95% CI 1.001-1.003, *P* < 0.001), CA125 (HR 1.035, 95% CI 1.007-1.063, *P* = 0.014), CD8 (HR 1.042, 95% CI 1.003-1.083, *P* = 0.036), and tumor location (HR 0.225, 95% CI 0.075-0.671, *P* = 0.007) were statistically significant. Multivariate analysis showed that the expression of FSIP1 (HR 0.143, 95% CI 0.069-0.295, *P* < 0.001), T classification (HR 0.109, 95% CI 0.033-0.357, *P* < 0.001), and N classification (HR 8.696, 95% CI 3.640-20.773, *P* < 0.001) were independent prognostic risk factors.

### 3.4. OS Prognosis Nomogram

According to Cox multivariate regression analysis, T classification, N classification, and the immunohistochemical expression level of FSIP1 were all independent prognostic factors in GC. The combination of these three factors in constructing a prediction nomogram model will help identify the death risk of 5 years in patients with GC (Figure 4(b)), which is helpful for evaluation of prognosis by the expression of FSIP1 more intuitively. Calibration curves showing a satisfactory applicability of nomogram model in predicting 3 and 5 years survival probability in GC patients, but poorly in predicting 2 years survival probability (Figure 4(c)).

## 4. Discussion

GC is a malignant tumor with high incidence and poor prognosis in developing countries. Its clinical manifestations are progressive digestive dysfunction and gastrointestinal obstruction. Individual heterogeneity in patients limits the therapeutic progress of GC. It is of great significance to study GC biomarkers for improving the survival probability and quality of life. Although FSIP1 is involved in a variety of biological reaction, its role in promoting the progression of GC remains unclear. Therefore, this study was aimed at evaluating the probability of FSIP1 as a prognostic predictor for GC.

FSIP1 is a fibrous sheath cytoskeletal protein and an essential protein involved in the formation of basic structure of elongated sperm cells [15]. Studies have found that FSIP1 and some other spermatogenesis-related genes are involved in the regulation of chromosome segregation [16]. Therefore, loss of FSIP1 leads to abnormal mitosis, including formation of multipolar spindles and prolonged mitotic interphase. Furthermore, FSIP1 was also found to be involved in sperm flagella formation and A-kinase anchor protein 4 (AKAP4) formation [17]. AKAP4 is one of the scaffold proteins associated with cAMP-dependent PKA and is highly expressed in various types of cancers [18, 19]. PKA and PKC play critical roles in tumor microenvironment angiogenesis, tumor cell transmembrane movement, and biological behavior [20, 21]. As a component of AKAP4, FSIP1 may play a role in tumor biology and thus may be a target for cancer immunotherapy.

In the field of tumor biology, FSIP1 is currently recognized as a cancer antigen which is highly expressed in breast, colon, lung, and bladder cancers and associated with poor prognosis. In breast cancer tissues, it has been found that FSIP1 expression is correlated with HER2 positive and Ki67 expression, and it is associated with poorer postoperative disease-specific survival probability [22]. Studies confirmed that FSIP1 directly binded to HER2 and inhibited the expression of multiple growth factors in HER2-positive breast cancer cells, leading to a decrease in cell proliferation, cell migration, and invasion capabilities and an increase in apoptosis. Due to its little expression in normal tissues, FSIP1 may become a potential drug target for the treatment of HER2-positive breast cancer. In addition, FSIP1 positively regulates proliferation and invasion of triple-negative breast cancer (TNBC) cells. It promotes drug resistance by inducing autophagy, reducing mitochondrial biosynthesis, and enhancing the activation of AMP-activated protein kinase [23]. In bladder cancer, FSIP1 knockdown inhibited the PI3K/AKT signaling pathway in vitro and in vivo, thereby suppressing the malignant behavior of bladder cancer cells. This suggests that targeting FSIP1 can be used for further potential therapeutic strategies of bladder cancer. However, the expression of FSIP1 in GC, its relationship with clinicopathological characteristics and prognosis, and its impact on related biological behaviors was rarely reported.

In this study, we evaluated the expression of FSIP1 in postoperative pathological tissue of 166 patients with GC and performed a statistical analysis of the follow-up data for at least 5 years. The results showed that the expression level of FSIP1 was significantly higher in GC tissues than that in paracarcinoma tissues. We analyzed the relationship between the expression level of FSIP1 and clinicopathological characteristics in patients with GC and found that the immunohistochemical expression of FSIP1 was an independent risk factor for the prognosis of advanced GC. Moreover, the calibration plot showed that the nomogram performed better in predicting 3-year and 5-year survival probability in GC patients, but poorly in predicting 2-year survival probability. This may be due to the limited sample size of patients and single-center study.

Our results indicate that the prediction model constructed by tumor T classification, N classification, and FSIP1 expression level can predict the prognosis of patients with GC. Similar models have been proposed in other various tumor studies, which is worthy of further validation and promotion in clinical application [24, 25]. Therefore, we speculate that FSIP1 may become a significant molecular marker for GC diagnosis, predicting prognosis and molecular targeted therapy, especially in patients with a younger age of onset or insensitive to chemotherapy drugs. However, we currently performed preliminary histological validation only in clinical samples. Further in vitro cell experiments were expected to explore the mechanism of FSIP1 acting on GC cells. These findings provide a basis for understanding the roles of FSIP1 processing the potential clinical implications of development of GC, which will provide a theoretical basis and new ideas for gene detection, diagnosis, and therapy target in the future.

## Figures and Tables

**Table 1:** The relationship between the expression of FSIP1 and clinicopathological characteristics.

Characteristic	High expression	Low expression	*P* value
Number of patients	63	103	
Age, mean ± SD	60.59 ± 9.59	57.56 ± 9.68	0.052
Weight, (median, IQR)	63 (55, 69.5)	65 (59.5, 72.5)	0.117
BMI, (median, IQR)	22.43 (19.58, 24.11)	23.03 (20.79, 24.93)	0.159
NLR, (median, IQR)	1.9 (1.3, 2.73)	1.95 (1.38, 2.6)	0.987
PLR, (median, IQR)	125.49 (100.25, 146.47)	116.75 (95.03, 151.9)	0.529
CD4, mean ± SD	36.15 ± 6.95	38.35 ± 8.07	0.075
CD8, (median, IQR)	23.3 (18.6, 29.85)	21.6 (16.95, 26.75)	0.170
CA19-9, (median, IQR)	10.32 (6.21, 19.96)	10.54 (6.21, 25.54)	0.874
CA72-4, (median, IQR)	3.65 (1.53, 6.55)	2.19 (1.37, 6.03)	0.105
CA125, (median, IQR)	10.15 (6.95, 14.34)	9.27 (6.52, 14.02)	0.592
^a^ Pathologic stage, *n* (%)			**< 0.001**
I	11 (6.6%)	47 (28.3%)	
II	17 (10.2%)	39 (23.5%)	
III	35 (21.1%)	17 (10.2%)	
Location, *n* (%)			**0.003**
L	42 (25.3%)	66 (39.8%)	
M	9 (5.4%)	32 (19.3%)	
U	10 (6%)	5 (3%)	
Entire	2 (1.2%)	0 (0%)	
WHO classification, *n* (%)			0.093
Mucinous	8 (4.8%)	5 (3%)	
Poor differentiated	10 (6%)	27 (16.3%)	
Signet ring cell	19 (11.4%)	22 (13.3%)	
Well to moderate differentiated	26 (15.7%)	49 (29.5%)	
Vascular invasion, *n* (%)			0.329
Negative	50 (30.1%)	89 (53.6%)	
Positive	13 (7.8%)	14 (8.4%)	
Neural invasion, *n* (%)			**0.012**
Negative	27 (16.3%)	66 (39.8%)	
Positive	36 (21.7%)	37 (22.3%)	

^a^ Based on the 8^th^ of the AJCC Cancer Staging Manual of the American Joint Committee on Cancer.

**Table 2:** Univariate and multivariate analyses of FSIP1 expression and clinicopathological characteristics for prognosis.

Characteristics	Total (*N*)	Univariate analysis		Multivariate analysis	
		Hazard ratio (95% CI)	*P* value	Hazard ratio (95% CI)	*P* value
FSIP1 expression	166				
High group	63	Reference			
Low group	103	0.143 (0.069-0.295)	**<0.001**	0.352 (0.156-0.796)	**0.012**
Age	166	1.031 (0.997-1.066)	0.072		
Sex	166				
Male	124	Reference			
Female	42	0.400 (0.156-1.026)	0.057		
^a^ T classification	166				
T2	25	Reference			
T3	78	0.109 (0.033-0.357)	**<0.001**	0.240 (0.061-0.945)	**0.041**
T1	53	0.078 (0.011-0.573)	**0.012**	0.201 (0.025-1.619)	0.132
T4	10	1.169 (0.411-3.325)	0.769	0.302 (0.067-1.355)	0.118
^b^ N classification	166				
N0	75	Reference			
N2	29	4.041 (1.504-10.854)	**0.006**	2.212 (0.769-6.363)	0.141
N3	35	8.696 (3.640-20.773)	**<0.001**	3.830 (1.306-11.231)	**0.014**
N1	27	1.178 (0.305-4.554)	0.813	0.667 (0.137-3.236)	0.615
BMI	166	0.921 (0.831-1.021)	0.118		
NLR	166	1.073 (0.899-1.280)	0.435		
PLR	166	1.003 (0.998-1.008)	0.217		
CD4	166	0.973 (0.933-1.015)	0.210		
CD8	166	1.042 (1.003-1.083)	**0.036**	1.044 (0.999-1.092)	0.054
CA19-9	166	1.002 (1.001-1.003)	**<0.001**	1.001 (1.000-1.003)	0.132
CA72-4	166	1.004 (0.996-1.012)	0.359		
CA125	166	1.035 (1.007-1.063)	**0.014**	1.023 (0.997-1.050)	0.085
Location	166				
U	15	Reference			
M	41	0.225 (0.075-0.671)	**0.007**	0.416 (0.130-1.337)	0.141
L	108	0.336 (0.144-0.786)	**0.012**	0.467 (0.169-1.287)	0.141
Entire	2	4.126 (0.829-20.534)	0.083	4.271 (0.675-27.019)	0.123
WHO classification	166				
Well to moderate differentiated	75	Reference			
Mucinous	13	2.404 (0.879-6.573)	0.087		
Poor differentiated	37	0.467 (0.156-1.396)	0.173		
Signet ring cell	41	1.643 (0.790-3.416)	0.184		
Vascular invasion	166				
Negative	139	Reference			
Positive	27	1.848 (0.874-3.907)	0.108		
Neural invasion	166				
Positive	73	Reference			
Negative	93	0.327 (0.167-0.639)	**0.001**	1.560 (0.609-3.995)	0.354

^ab^ Based on the 8^th^ of the AJCC Cancer Staging Manual of the American Joint Committee on Cancer.

## Data Availability

The datasets used in this study are available from the corresponding author on reasonable request.
